# The Selective Antagonism of P2X_7_ and P2Y_1_ Receptors Prevents Synaptic Failure and Affects Cell Proliferation Induced by Oxygen and Glucose Deprivation in Rat Dentate Gyrus

**DOI:** 10.1371/journal.pone.0115273

**Published:** 2014-12-19

**Authors:** Giovanna Maraula, Daniele Lana, Elisabetta Coppi, Francesca Gentile, Tommaso Mello, Alessia Melani, Andrea Galli, Maria Grazia Giovannini, Felicita Pedata, Anna Maria Pugliese

**Affiliations:** 1 Dept. of Neuroscience, Psychology, Drug Research and Child Health, NEUROFARBA, Division of Pharmacology and Toxicology, University of Florence, Florence, Italy; 2 Dept. of Health Sciences, Clinical Pharmacology and Oncology Unit, University of Florence, Florence, Italy; 3 Dept. of Experimental and Clinical Biomedical Sciences, University of Florence, Florence, Italy; Karolinska Inst, Sweden

## Abstract

Purinergic P2X and P2Y receptors are broadly expressed on both neurons and glial cells in the central nervous system (CNS), including dentate gyrus (DG). The aim of this research was to determine the synaptic and proliferative response of the DG to severe oxygen and glucose deprivation (OGD) in acute rat hippocampal slices and to investigate the contribution of P2X_7_ and P2Y_1_ receptor antagonism to recovery of synaptic activity after OGD. Extracellular field excitatory post-synaptic potentials (fEPSPs) in granule cells of the DG were recorded from rat hippocampal slices. Nine-min OGD elicited an irreversible loss of fEPSP and was invariably followed by the appearance of anoxic depolarization (AD). Application of MRS2179 (selective antagonist of P2Y_1_ receptor) and BBG (selective antagonist of P2X_7_ receptor), before and during OGD, prevented AD appearance and allowed a significant recovery of neurotransmission after 9-min OGD. The effects of 9-min OGD on proliferation and maturation of cells localized in the subgranular zone (SGZ) of slices prepared from rats treated with 5-Bromo-2′-deoxyuridine (BrdU) were investigated. Slices were further incubated with an immature neuron marker, doublecortin (DCX). The number of BrdU^+^ cells in the SGZ was significantly decreased 6 hours after OGD. This effect was antagonized by BBG, but not by MRS2179. Twenty-four hours after 9-min OGD, the number of BrdU^+^ cells returned to control values and a significant increase of DCX immunofluorescence was observed. This phenomenon was still evident when BBG, but not MRS2179, was applied during OGD. Furthermore, the P2Y_1_ antagonist reduced the number of BrdU^+^ cells at this time. The data demonstrate that P2X_7_ and P2Y_1_ activation contributes to early damage induced by OGD in the DG. At later stages after the insult, P2Y_1_ receptors might play an additional and different role in promoting cell proliferation and maturation in the DG.

## Introduction

The hippocampus comprises two distinct subfields that show different responses to hypoxic-ischemic brain injury. The CA1 region is particularly susceptible to hypoxia, whereas the dentate gyrus (DG), which serves as a gateway to the hippocampus, is usually more resistant [1]. We have recently demonstrated that in the DG it is necessary to prolong OGD duration to 9 min in order to consistently induce the appearance of anoxic depolarization (AD) and synaptic depression, whereas in the CA1 area 7 min are sufficient [2]. The generation of AD is complex and multifactorial (see: [3]). After OGD initiation, the large efflux of K^+^ ions into the extracellular space, combined with activation of Na^+^ and Ca^2+^ channels, triggers sustained depolarization of hippocampal cells that coincides with the appearance of AD. Increased intracellular Ca^2+^ and/or massive glutamate receptor activation are additional mechanisms that concur to produce AD [4], [5] and that contribute to cell damage during ischemia [3]. A delay in the appearance of AD can be obtained by treating the slices with glutamate receptor antagonists [4], [5].

A major resistance of the DG to ischemia in adulthood [1] is probably due to its regenerative capacity [6], [7], [8]. It is indeed known that adult neurogenesis persists in two restricted regions of the mammalian brain: the subventricular zone (SVZ) of the lateral ventricle (LV; [9]) and the subgranular zone (SGZ) in the hippocampal DG [7], [10]. These neurogenic niches provide microenvironments that regulate the proliferation and differentiation of neural stem cells [11]–[15]. These cells are able to proliferate and differentiate into neurons, astrocytes and oligodendrocytes [16] in response to multiple factors, including hypoxic-ischemic injury [17], [18], [1]. An increase in DG cell proliferation has been demonstrated in different animal models of brain ischemia *in vivo* [17], [1] or in oxygen–glucose-deprived hippocampal slice cultures [19]. Recently, we have demonstrated in acutely isolated hippocampal slices, the presence of proliferating neuronal progenitor cells in the SGZ of the DG, whose maturation is promoted by a severe OGD [2].

The role of ATP in cerebral ischemia has been studied in the last decade [20]. During ischemia, ATP intracellular concentrations decline [21] to refurnish energy to cells. However, in this condition ATP outflow from cells increases, as demonstrated *in vivo* [22], [23], in *ex vivo* brain slices [24] and in *in vitro* cell cultures [17], [25]. Extracellularly, ATP acts on P2 receptors that are subdivided into ligand-gated ion channels, P2X, and metabotropic P2Y receptors [26], [27]. Several data including ours [28], [29] highlight the involvement of P2X_7_ [30] and P2Y_1_ subtypes [31]–[34] in the control of ischemic brain damage.

P2X_7_ receptor (P2X7R) expression, initially identified in glial cells in the CNS [35], [36], has been later found expressed on neurons in the brain, including the hippocampal area [37]–[40]. P2X7R mRNA has also been found in the SGZ of DG from E18.5 to adulthood where it colocalizes with a marker of immature neurons [41].

The P2Y1 receptor (P2Y1R) is widely distributed throughout rat brain including hippocampus, both on neurons and glial cells [42]–[45]. We have recently demonstrated that in the CA1 region of hippocampal slices, the selective block of P2Y1R and P2X7R antagonizes the depression of synaptic potentials induced by a severe OGD period, delays the appearance of AD [28], [46] and protects from the CA1 injury assessed by propidium iodide staining [46]. These results suggest that the selective antagonism of P2X7R and P2Y1R may be an effective strategy to improve cell survival and function after OGD.

So far no data are available on the role of these receptors on neurotransmission and proliferative response in the DG, before and after an ischemic insult. Thus, the purpose of our research was to study the contribution of P2X7R and P2Y1R to the recovery of neurotransmission and to the modulation of proliferative and maturational responses in the DG in acutely isolated hippocampal slices.

## Materials and Methods

### In vivo BrdU treatments

All animal procedures were conducted according to the Italian Guidelines for Animal Care, DL 116/92, application of the European Communities Council Directive (86 / 609 / EEC). Experiments were approved by the Institutional Animal Care and Use Committee (IACUC) of the University of Florence and performed according to the Italian Law on Animal Welfare (DL 116/92). All efforts were made to minimize animal sufferings and to use only the number of animals necessary to produce reliable scientific data.

Male Wistar rats (Harlan Italy; Udine Italy, 150–200 g body weight) were used. Two intraperitoneal injections (i.p.) of BrdU (50 mg/kg) were given with the interval of 6 h for three consecutive days. BrdU was dissolved in saline solution (sterile 0.9% NaCl) and prepared freshly every day of injection. After 24 hours from last injection, the animal was sacrificed and hippocampal slices prepared as described below.

### In vitro experiments

#### Slice preparation

Experiments were carried out on acute hippocampal slices [2], [46], Animals were killed with a guillotine under anesthesia with isoflurane (Baxter, Rome, Italy) and their hippocampi were rapidly removed and placed in ice-cold oxygenated (95% O_2_–5% CO_2_) artificial cerebrospinal Fluid (aCSF) of the following composition (mM): NaCl 125, KCl 3, NaH_2_PO_4_ 1.25, MgSO_4_ 1, CaCl_2_ 2, NaHCO_3_ 25 and D-glucose 10. Sagittal brain slices (400 µm), composed of hippocampus and overlying neocortex, were then cut in ice-cold, (400 µm thick) using a McIlwain tissue chopper (The Mickle Lab. Engineering, Co. Ltd., Gomshall, U.K.) and kept in oxygenated aCSF for at least 1 hour at room temperature (RT). A single slice was then placed on a nylon mesh, completely submerged in a small chamber (0.8 ml) and superfused with oxygenated aCSF (31–32°C) at a constant flow rate of 1.5–1.8 ml min^−1^. Changes in superfusing solutions (OGD or drugs) reached the preparation in 60 s and this delay was taken into account in our calculations.

#### Extracellular recording

The field potential recordings were performed in the mid-molecular layer of DG following stimulation of the perforant pathway. Test pulses (80 ms, 0.066 Hz) were delivered through a bipolar nichrome electrode positioned into the perforant pathway for DG recordings. Evoked extracellular potentials were recorded with glass microelectrodes (2–10 MΩ, Harvard Apparatus LTD, Edenbridge, UK) filled with 150 mM NaCl and placed into the mid-DG. Test stimuli were delivered to perforant pathway every 15s. Responses were amplified (200×, BM 622, Mangoni, Pisa, Italy), digitized (sample rate, 33.33 kHz), and stored for later analysis with LTP (version 2.30D) program [47].

Stimulus-response curves were obtained by gradual increases in stimulus strength at the beginning of each experiment, when a stable baseline of evoked response was reached. The test stimulus pulse was then adjusted to produce a field Excitatory Post Synaptic Potential (fEPSP) whose slope and amplitude was 40% to 50% of the maximum and was kept constant throughout the experiment. The fEPSP amplitude was routinely measured and expressed as the percentage of the average amplitude of the potentials measured during the 5 min preceding exposure of the hippocampal slices to OGD. In some experiments, both the amplitude and initial fEPSP slope were quantified, but because no appreciable differences between these two parameters were observed in drug effect and OGD, only the amplitude measurement is expressed in the Figures. Simultaneously with fEPSP amplitude, AD, induced by 9 or 30 min OGD, was recorded as negative extracellular direct current (d.c.) shifts. The d.c. potential is an extracellular recording considered to provide an index of the polarization of cells surrounding the tip of the glass electrode [48]. AD latency, expressed in min, was calculated from the beginning of OGD; AD amplitude, expressed in mV, was calculated at the maximal negativity peak. In the text and bar graphs, AD amplitude values were expressed as positive values.

Conditions of OGD were obtained by superfusing the slice with aCSF without glucose and gassed with nitrogen (95% N_2_–5% CO_2_) [49]. This caused a drop in pO_2_ in the recording chamber from ∼500 mmHg (normoxia) to a range of 35–75 mmHg (after 7 min OGD) [50]. At the end of the ischemic period, the slice was again superfused with normal, glucose-containing, oxygenated aCSF. Throughout this paper, the terms ‘untreated OGD slices’ or ‘treated OGD slices’ refer to hippocampal slices in which OGD episodes of different duration were applied in the absence or in the presence of drugs, respectively. Control slices were not subjected to OGD or drug treatment, but were incubated in oxygenated aCSF for identical time intervals. When we applied P2 antagonists (for 24 min) under normoxic conditions, each slice was fixed 6 or 24 hours after treatment.

In the present study, we used two P2 antagonists: 2′-Deoxy-*N* ^6^-methyladenosine 3′,5′-bisphosphate (MRS2179) and Brilliant Blue G (BBG). MRS2179 is a competitive antagonist at P2Y_1_ receptors (K_B_ = 100 nM) [51]. The concentration of MRS2179 used in our experiment was chosen according to the literature [28]. BBG is the most used antagonist for P2X7R, with apparent IC_50_ values of 0.01–0.2 µM [52], [53]. The concentration of BBG used in this work was chosen on the basis of our previous experiments conducted in the CA1 region [28], [46].

### Immunohistochemical assay

Proliferating cells were detected by using the DNA replication marker 5-Bromo-2′-deoxyuridine (BrdU), a thymidine analogue which incorporates into the DNA of all cells during the S-phase. To determine the phenotype of the newly born cells, doublecortin (DCX), an immature neuronal marker, was used. DCX is a cytoskeleton-associated protein that is transiently expressed during adult neurogenesis. Antibodies against DCX are among the most widely used markers for detection of neuronal progenitors and identify type-2b and type-3 cells, which belong to blast-like late progenitors and exhibit the first morphological signs of neuronal maturation [54]–[56]. DCX is expressed in newborn hippocampal granule cells during the first 3 weeks after mitosis [55].

#### BrdU and DCX staining

After extracellular recordings, slices were maintained in separate chambers in oxygenated aCSF at RT before fixing, which was performed at different time points: after 3, 6 or 24 hours from the end of OGD. Control slices were monitored for electrophysiological activity and fixed at corresponding times after slicing. Hippocampal slices (400 µm) were then fixed overnight using 1 ml of 4% ice-cold paraformaldehyde and then cryoprotected in a sucrose solution (18%) for at least 48 hours. Then, slices were glued on frozen cubes of agar (4%), prepared in 18% sucrose solution, and then re-sliced into 50-µm thick slices with a cryostat. External slices from each side were excluded, while one slice taken from the inner part was placed in antifreeze solution (30% ethylene glycol, 30% glycerol in phosphate buffer) at −20°C, until immunohistochemical assay.

*Day 1*. Slices were stained using the free-floating method described by Giovannini et al. [57], [58]. Hippocampal slices were placed in 24-well plates and rinsed for 10 min in phosphate-buffered saline–0.3% Triton X-100 (PBS-TX). Briefly, DNA denaturation was achieved by treatment with 2 M HCl at 36°C for 15 min and then rinsed in borate buffer for 10 min (0.1 M, pH 8.5 at RT). They were extensively washed with PBS containing 0.3% Triton X-100 and then incubated on a shaker for 1 hour at RT with PBS containing 0.3% Triton X-100, 0.05% NaN_3_, 10% normal goat serum and 10% normal horse serum (blocking solution). For double-labelling experiments, after a PBS solution containing 0.3% Triton X-100 rinse, sections were incubated with at least 250 µl in mouse monoclonal anti-BrdU antibody (1∶300; Abcam, Cambridge, UK) and rabbit monoclonal anti-DCX antibody (1∶500; Abcam, Cambridge, UK) diluted in blocking solution, overnight at 4°C on a shaker.

*Day 2.* Primary antibodies were removed and slices were washed several times with PBS solution containing 0.3% Triton X-100. From this step, all procedures were carried out in the dark. Sections were then incubated under agitation for 2 hours at RT with a horse anti-mouse fluorescent secondary Antibody (Fluorescein Anti-Mouse IgG, 1∶500, Vector laboratories, USA) dissolved in blocking buffer. The secondary antibody was then removed and the slices were washed several times with PBS solution containing 0.3% Triton X-100 and incubated at RT with a goat anti-rabbit fluorescent secondary antibody (Alexa Fluor 635, 1∶500, Invitrogen Ltd, UK) dissolved in blocking buffer. After 2 hours of incubation at RT, the secondary antibody was removed and slices were washed with PBS solution 0.3% Triton X-100. Finally, after an extensive washout with distillate water, slices were mounted onto gelatin-coated slides for microscopic examination using Pro-Long mounting medium (Invitrogen Ltd, UK).

Image analysis of double immunofluorescent labelling was performed by a SP2-AOBS confocal laser-scanning microscope (Leica Microsystems, Mannheim, Germany) through a 20X 0.5NA air objective and 40X 1.2NA oil-immersion objective, using laser excitation at 488 and 635 nm. Images were acquired as Z-stacks of the entire slice thickness (50 µm), with sampling at 1.5-µm (20X) or 0.3 µm (40X) intervals for the entire slice depth (50 µm) and assembled into montages with Image J software (NIH; http://rsb.info.nih.gov/ij/) and Adobe Photoshop 7.0 (Adobe Systems, Mountain View, CA, USA). As already described [2] double-labelled cells were quantified in a confocal plane throughout the area (750×750 µm), captured at 20× magnification, and the total number of BrdU^+^ cells were counted by eye in the SGZ. In particular, all quantification analysis were performed blind by two different experimenter and results were averaged. Only those cells that were found within the granule cell layer, in the SGZ, defined as the 20-µm band under the granule cell layer were included in the analysis. The number of experiment used for the quantification analysis corresponds to the number of slices used. Each treated slice was taken from a different rat and had a corresponding control. In most experiments we used the middle or the dorsal portion of the hippocampus since it is known to be most severely and consistently affected by ischemia [59].

DCX immunofluorescence analysis: ten confocal z-scans (each 1.5 µm, total 15 µm) of DCX-labelled slices (red channel, Alexa Fluor 635), captured at 20× magnification were stacked and thresholded throughout the area (750×750 µm) using the Threshold tool of ImageJ software. Care was taken to maintain the same threshold value in control and treated slices. The area above the set threshold was calculated in pixels (using Histogram Tool) and the data were then analyzed using GraphPad Prism 5.0 (GraphPad Software, Inc. S. Diego, CA).

### Drugs

MRS2179 was purchased from Tocris (Bristol, UK). BBG and BrdU were purchased from Sigma (Sigma-Aldrich, Italy). Both P2 purinergic receptor antagonists were dissolved in distilled water and applied 10 min before, during and 5 min after 9 min of OGD. Stock solutions of 100–10,000 times the desired final concentration were stored at −20°C.

### Statistics

Statistical significance was evaluated by Student's paired or unpaired *t* tests. Analysis of variance (one-way ANOVA), followed by Newman–Keuls multiple comparison *post hoc* test was also used, as appropriate. *P*-values from both Student's paired and unpaired *t* tests are two-tailed. Data were analysed using software package GraphPad Prism (version 5.0; GraphPad Software, San Diego, CA, USA). All numerical data are expressed as the mean±standard error of the mean (SEM).

## Results

The role of P2X7R and P2Y1R stimulation by endogenous ATP released during OGD episodes on DG synaptic transmission was investigated using selective P2X7R and P2Y1R antagonists. Electrically evoked fEPSPs were extracellularly recorded in the DG of 140 hippocampal slices taken from 53 rats for monitoring the time course of the effects of OGD episodes on synaptic responses, both in control and treated slices.

In a subset of experiments, after electrophysiological recordings, each slice was subjected to immunohystochemical analysis.

### Involvement of P2X7R and P2Y1R in synaptic failure induced by severe OGD in DG

**Figure 1:**
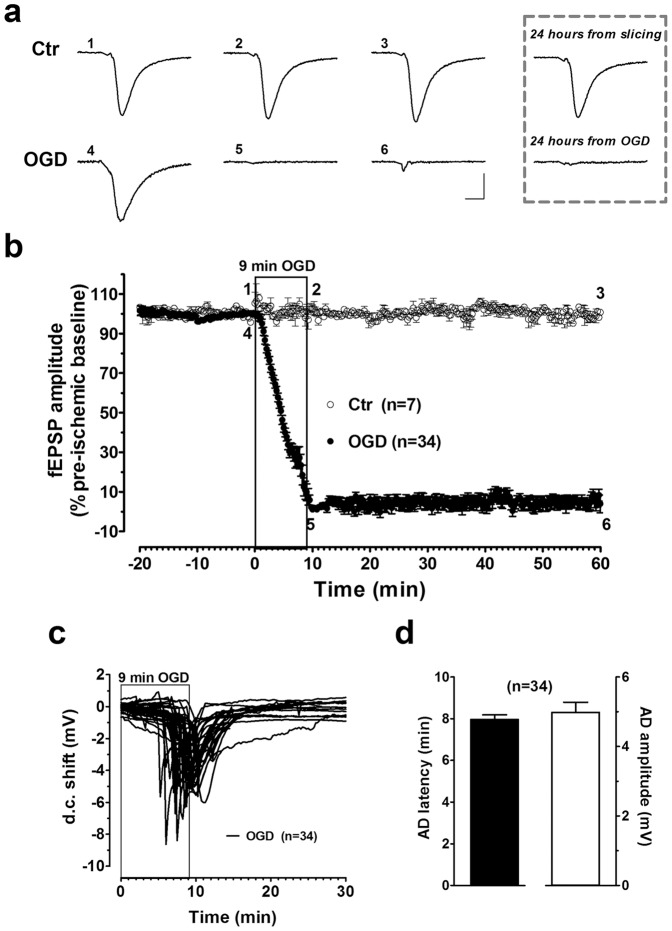
AD development and synaptic failure in the DG of rat hippocampal slices after 9-min OGD. **a**. Upper panel: fEPSP taken from a typical experiment at the time-points indicated on the graph in a control hippocampal slice (Ctr) continuously superfused with oxygenated aCSF. Lower panel: example traces taken at the time-points indicated on the graph recorded from a hippocampal slice before (4), at the end of 9-min OGD (5) or 50 min after washout in oxygenated aCSF (6). **b**. The graph shows the time-course of the fEPSP amplitude, expressed as percent of pre-ischemic baseline in the DG (mean±SEM) in a group of control slices (Ctr, n = 7) or in a group of slices subjected to 9-min OGD (OGD, n = 34). Note that, while a stable fEPSP was recorded in control slices, the ischemic insult elicited a gradual reduction of fEPSPs amplitude, which completely disappears and does not recover even after prolonged washing in oxygenated aCSF. Inset: example traces taken from the same respective hippocampal slices shown in panel **a**, in control (Ctr, recorded 24 hours from slice preparation) or 24 hours from the end of OGD. In each graph, traces are averages of 3 consecutive responses. Scale bars: 10 ms, 0.5 mV. **c**. Anoxic depolarization (AD) was recorded as a negative d.c. shift in response to 9-min OGD. The d.c. shift was always recorded (n = 34) during 9-min OGD. **d**. Each column represents the mean±SEM of AD latency or AD amplitude recorded in the DG during 9-min OGD. AD latency was measured from the beginning of OGD insult.

In a first series of experiments we characterized the synaptic response of evoked fEPSPs in the DG in the absence or following severe (9-min) OGD, an ischemic insult that under our experimental conditions constantly produces an irreversible loss of synaptic transmission [2]. At the same time, we also monitored the d.c. shift produced by AD, a further important parameter of brain tissue integrity [3]. According to our previous works [49], [2], hippocampal slices, as thick as 400 µm, remained viable for several hours after slicing and a steady fEPSP was monitored for up to 1 hour or more (Fig. 1a,b). In these conditions, no change in d.c. traces was recorded (not shown). A 9-min OGD episode induced the disappearance of the fEPSP, which did not recover (4.0±5.0%, n = 34, Fig. 1a,b) after superfusion with oxygenated, glucose-containing aCSF, as monitored up to 24 hours from the end of OGD (inset of Fig. 1a shows a representative fEPSP; similar results were obtained in 12 other slices, not shown). In all slices subjected to OGD, AD was recorded as a voltage shift with a mean peak latency of 8.0±0.2 min (n = 34, Fig. 1c,d) after OGD initiation and a mean peak amplitude of 5.0±0.3 mV (n = 34, Fig. 1c,d). Therefore, it is evident that an OGD time duration of 9 min is a severe insult for the DG.

The effect of P2 purinergic receptor stimulation by endogenous ATP released during OGD episodes on DG synaptic transmission was investigated using two different P2 purinergic selective antagonists.

**Figure 2:**
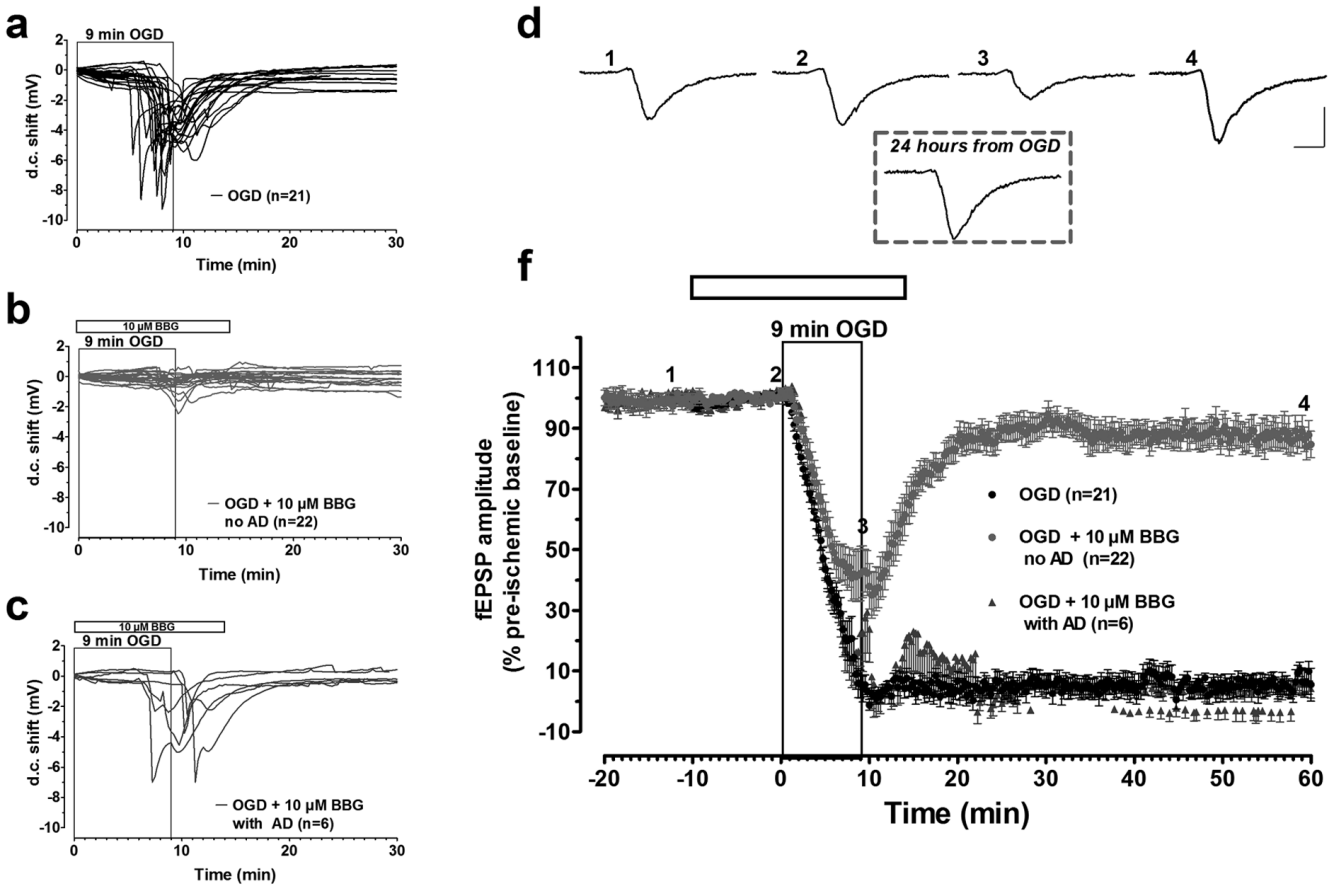
The P2X7R antagonist, BBG, prevents the synaptic failure induced by 9-min OGD in the DG. AD was recorded as a negative d.c. shift in response to 9-min OGD in control conditions (**a**, n = 21). BBG (10 µM) completely prevented the appearance of AD in 22 out of 28 slices during 9-min OGD (**b**). BBG (10 µM) had no effect in 6 out of 28 slices after 9-min OGD (**c**, n = 6), in which AD was recorded as a negative d.c. shift. **d**. example traces taken at the time-points indicated on the graph, immediately before (1), 10 min after the beginning of BBG application (2), 9 minutes after OGD+BBG (3) or 50 min after washout in oxygenated aCSF (4). Inset: example trace taken from the same hippocampal slice 24 hours from the end of OGD applied in the presence of 10 µM BBG. In each graph, traces are averages of 3 consecutive responses. Scale bars: 10 ms, 0.5 mV. **f**. The graph shows the time-course of the effect caused by 9-min OGD on fEPSP amplitude (mean ± SEM) in untreated OGD slices (n = 21) and in OGD slices treated with 10 µM BBG, in which AD was absent (n = 22) or present (n = 6). fEPSPs amplitude is expressed as percent of the respective pre-ischemic baseline. Open bar: time of drug application.

As shown in Fig. 2a, in untreated slices, 9-min OGD always caused the appearance of AD. BBG, the most widely used selective P2X7R antagonist, applied 10 min before, during and 5 min after the OGD, completely prevented the AD appearance induced by 9-min OGD in 22 out of the 28 slices tested (Fig. 2b). In these 22 slices, BBG (10 µM) allowed a significant recovery of fEPSP amplitude within 15-min of reperfusion with oxygenated and glucose-containing aCSF (84.7±4.3% in comparison to 5.5±5.4% found in OGD-untreated slices, n = 21, calculated after 50 min from the end of OGD, Fig. 2d,f). The recovery of fEPSP was maintained up to 24 hours from the end of OGD (inset of Fig. 2d shows a representative fEPSP; similar results were obtained in 4 other slices). Fig. 2c shows that only 6 out of 28 slices treated with 10 µM BBG and exposed to 9-min OGD presented a sizeable AD (>1 mV) whose peak latency was on average delayed (8.9±0.8 min, n = 6). No changes in AD mean amplitude were observed (4.4±0.6 mV, n = 6). On average, in these 6 slices, no fEPSP recovery was observed (4.3±4.2% of the pre-ischemic baseline, Fig. 2f). In addition, during the first 2 min of recovery from the 9-min OGD episode, a transient reappearance of the fEPSP was observed (Fig. 2f), caused by the rise in extracellular K^+^ concentration [60]. However, one of these 6 slices, in spite of the appearance of AD (2.5 mV, 110 s after oxygen and glucose restoration) recovered a significant fraction (38.5%) of original fEPSP amplitude after 20 min of reperfusion (not shown). On the bases of these results, we can conclude that in the DG the P2X7R antagonist was effective in protecting from a severe OGD insult in 79% of the slices tested.

**Figure 3:**
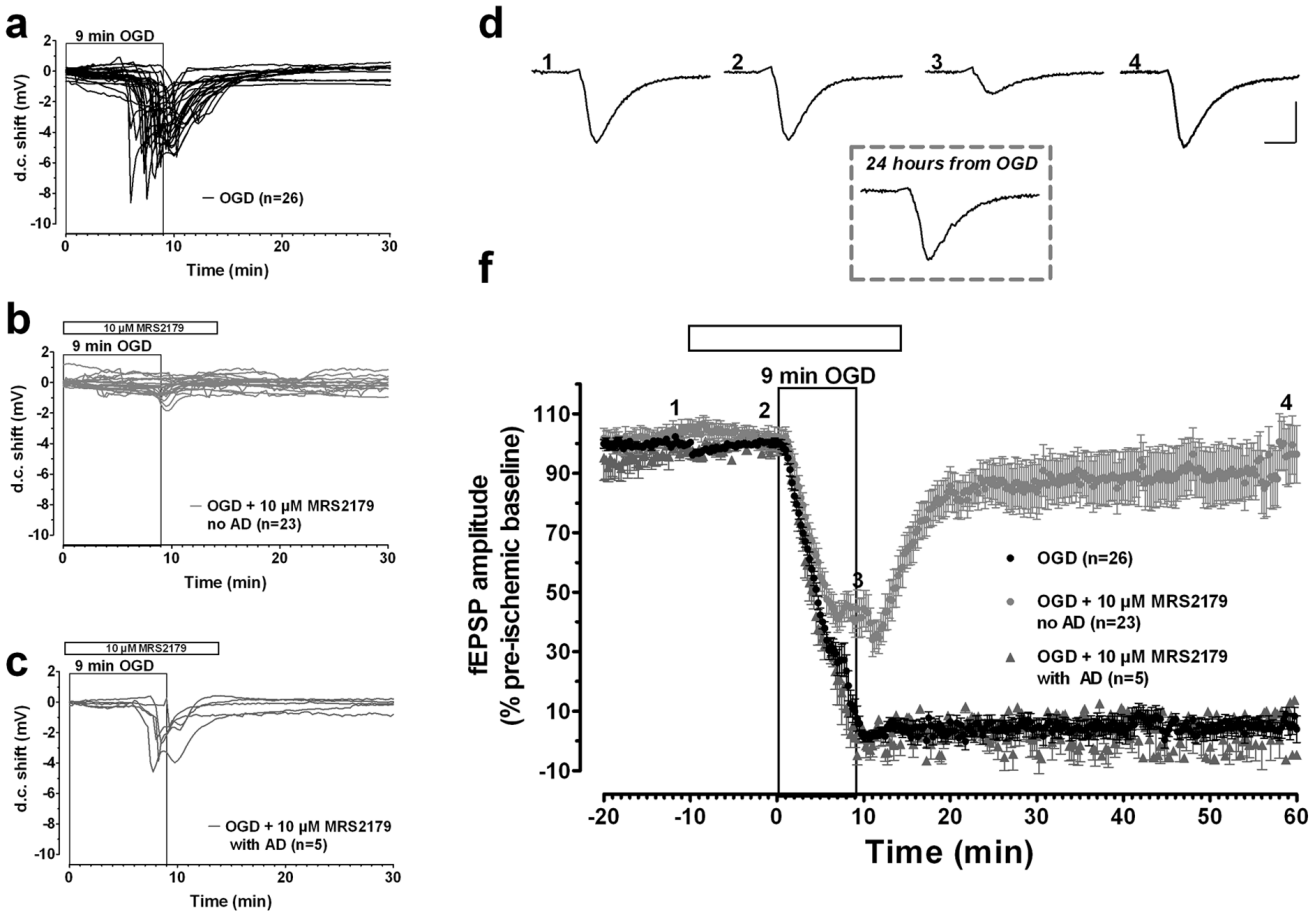
The block of P2Y1R counteracts the synaptic failure induced by severe OGD in the DG. AD was recorded as a negative d.c. shift in response to 9-min OGD in control (**a**, n = 26). MRS2179 (10 µM) completely prevented the appearance of AD in 23 out of 28 slices during 9-min OGD (**b**). MRS2179 (10 µM) had no effect in 5 out of 28 slices after 9-min OGD (**c**, n = 6), in which AD was recorded as a negative d.c. shift. **d**. example traces taken at the time-points indicated on the graph, immediately before (1), 10 min after the beginning of MRS2179 application (2), 9 minutes after OGD+MRS2179 (3) or 50 min after washout in oxygenated aCSF (4). Inset: example trace taken from the same hippocampal slice 24 hours from the end of OGD applied in the presence of 10 µM MRS2179. In each graph, traces are averages of 3 consecutive responses. Scale bars: 10 ms, 0.5 mV. **f**. The graph shows the time-course of the effect caused by 9-min OGD on fEPSP amplitude (mean±SEM) in untreated OGD slices (n = 26) and in OGD slices treated with 10 µM MRS2179, in which AD was absent (n = 23) or present (n = 5). fEPSPs amplitude is expressed as percent of the respective pre-ischemic baseline. Open bar: time of drug application.

As shown in Fig. 3, similar results were obtained in the presence of MRS2179, a selective P2Y1R antagonist, applied 10 min before, during and 5 min after the OGD. MRS2179 (10 µM, n = 28) prevented the appearance of AD in 23 out of 28 slices tested (Fig. 3b). In these 23 slices, in which AD was absent, we observed an almost complete fEPSP recovery of 96.0±12.5%, calculated 50 min from the end of OGD, in comparison to that obtained in the absence of the drug (4.0±4.5%, n = 26, Fig. 3d,f). The recovery of synaptic potentials was maintained up to 24 hours from the end of OGD (inset of Fig. 3d shows a representative fEPSP; similar results were obtained in 6 other slices). In the remaining 5 out of 28 slices, MRS2179 was not effective, since neither changes on AD appearance (Fig. 3c) nor fEPSP recovery (9.0±1.1%) were detected (Fig. 3f). In these 5 slices AD peaked at 8.1±0.2 min, with a mean amplitude of 4.4±0.6 mV. On the bases of these results, we can conclude that in the DG the P2Y1R antagonist was effective in 82% of the slices tested.

**Figure 4:**
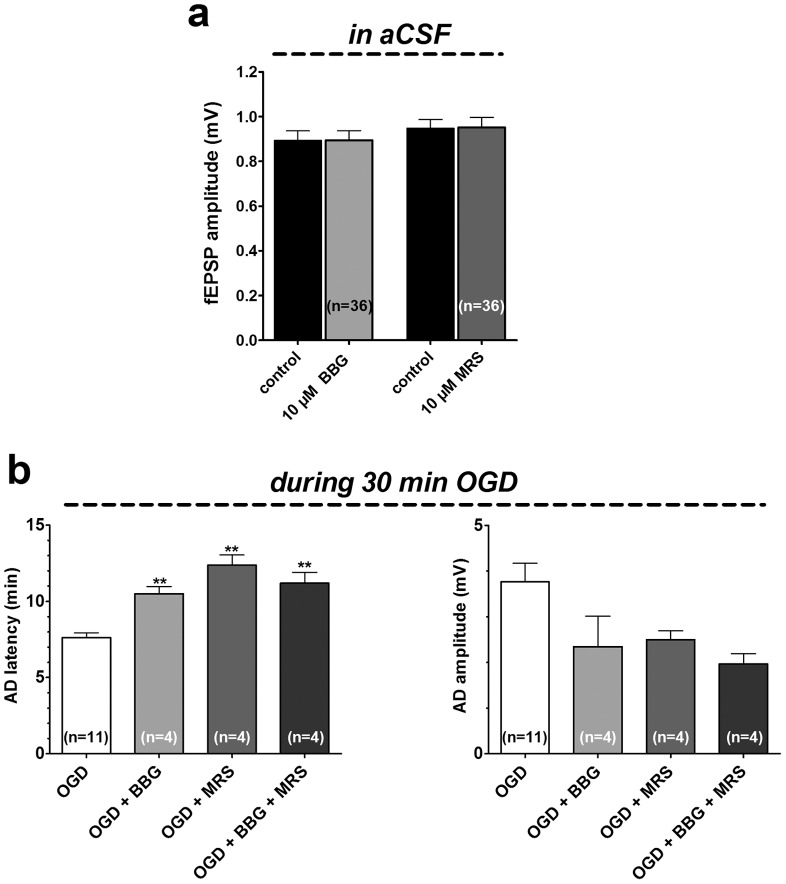
Antagonism of P2X7R or P2Y1R does not affect basal synaptic transmission, but significantly delays the AD appearance during prolonged OGD. a. Bars in the graph represent the average fEPSP amplitude (mean±SEM) recorded immediately before (control) and at the end of 10 min application of 10 µM BBG or 10 µM MRS2179. b. Each column represents the mean ± SEM of AD latency (left panel) and AD amplitude (right panel) recorded in the DG during 30 min OGD in the absence (n = 11) or in the presence of BBG (10 µM, n = 4) or MRS2179 (10 µM, n = 4) alone or in combination (n = 4). Note that both P2 antagonists significantly delayed AD development and that this effect is not modified by the combination of the two drugs (**P<0.01, One-way ANOVA followed by Newman–Keuls post hoc test, compared to untreated OGD slices). AD latency was measured from the beginning of OGD insult. The number (n) of slices tested is reported inside columns.

BBG (10 µM, n = 36) and MRS2179 (10 µM, n = 36) did not change fEPSP amplitude under basal, normoxic, conditions. The fEPSP values ranged from 0.89±0.05 mV before BBG to 0.90±0.04 mV, n = 36, calculated after 10 min BBG application, (Fig. 2f and Fig. 4a) and from 0.95±0.04 mV before MRS2179 to 0.96±0.04 mV, n = 36, calculated at the end of 10 min MRS2179 application (Fig. 3f and Fig. 4a).

Finally, in order to better characterize the effects of BBG and MRS2179 on AD development and to verify if the two antagonists, applied together, might also have additive effects or produce different outcome, we prolonged the duration of the OGD from 7 to 30 min. This longer duration of OGD is invariably associated with tissue damage [2]. We compared the time of appearance and the magnitude of depolarizing d.c. shift, in the absence or in the presence of the P2 receptor antagonists, alone or in combination. As illustrated in Fig. 4b, 30 min OGD elicited the appearance of AD in untreated OGD slices, with a mean peak latency of 7.6±0.3 min and a mean amplitude of 3.8±0.4 mV (n = 11). When 30 min OGD was applied in the presence of 10 µM BBG or 10 µM MRS2179 or during co-application of both compounds, the d.c. shifts were always delayed (Fig. 4b) and no additive effect was observed. In particular, AD latency was: 10.5±0.5 min in the presence of 10 µM BBG, n = 4; 12.4±0.7 min in 10 µM MRS2179, n = 4; 11.2±0.7 min, n = 4 in the presence of 10 µM BBG and 10 µM MRS2179, n = 4. Conversely, AD amplitude was not significantly changed (2.4±0.7 mV in the presence of 10 µM BBG, n = 4; 2.5±0.2 mV in 10 µM MRS2179, n = 4; 2.0±0.2 mV in the presence of 10 µM BBG and 10 µM MRS2179, n = 4, Fig. 4b).

### Severe OGD affects early phase of cell proliferation and maturation in the SGZ of DG: role of P2X7R and P2Y1R

**Figure 5:**
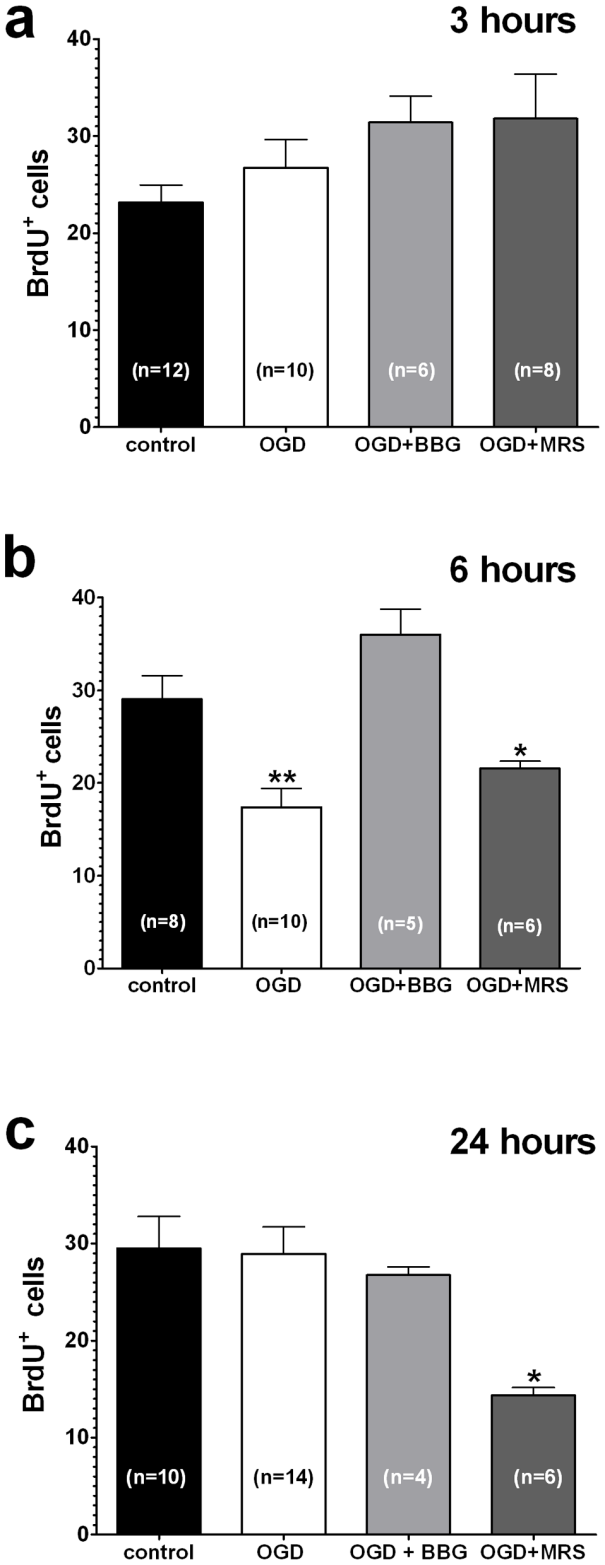
Temporal profile of cell proliferation in the SGZ of DG. Quantification of BrdU^+^ cells in the SGZ of the DG at 3, 6 and 24 hours after the end of OGD. Each column shows the total number of BrdU^+^ cells in the SGZ. Bars represent the mean±SEM. In parentheses is the number of slices investigated. *P<0.05 and **P<0.01 *vs* control, One-way ANOVA followed by Newman–Keuls post hoc test.

**Figure 6:**
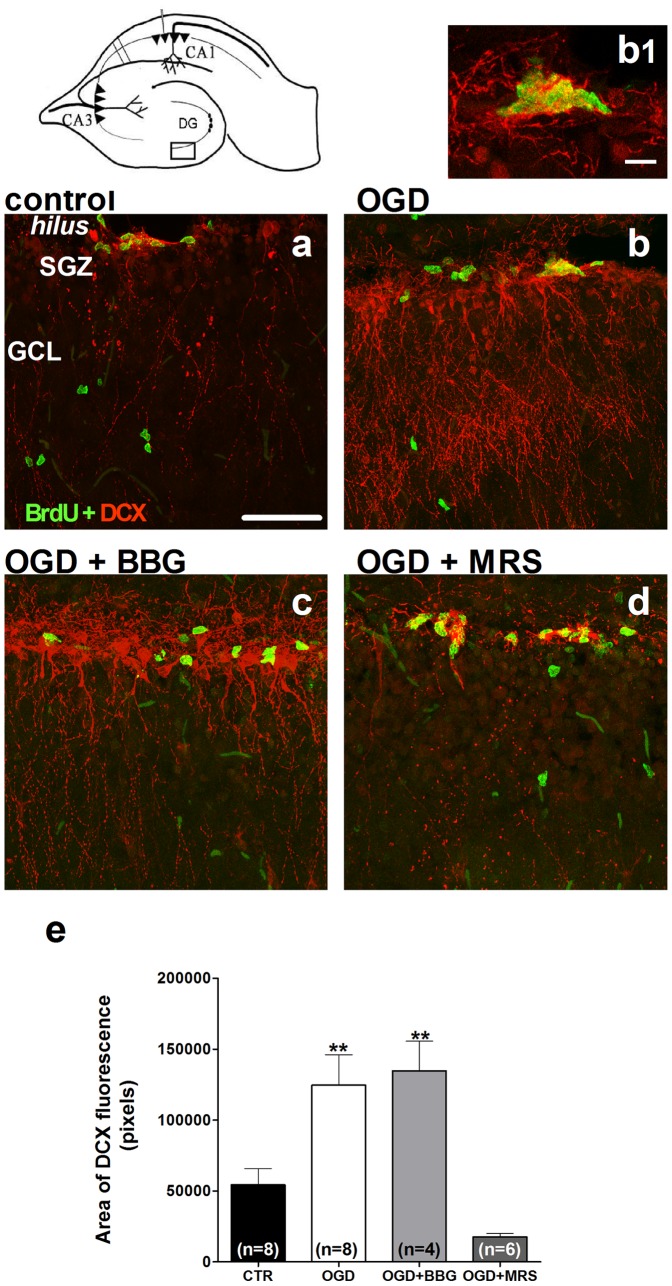
Nine-min OGD induces an increase of DCX immunofluorescence in the SGZ of DG; this effect is antagonized by MRS2179. Immunohistochemical staining of BrdU^+^ and DCX^+^ cells in the SGZ of DG. Upper left panel: a schematic hippocampal slice. The region in the box (black arrow) represents the SGZ shown in a-d. Panels a-d: Confocal images of the double-staining immunohistochemical analysis of BrdU^+^ (green) and DCX^+^ (red) cells in the SGZ. Double-stained cells are clearly visible in the SGZ of DG in control slices 24 hours after slice preparation (a), and 24 hours from the end of OGD carried out in the absence (b) or in the presence of 10 µM BBG (c) or 10 µM MRS2179 (d). DCX^+^ neuroblasts show higher DCX immunofluorescence, if compared to control slice, both after OGD alone or OGD+BBG. This effect was not evident in the slice in which OGD was applied in combination with MRS2179 (d). The vast majority of BrdU^+^ nuclei belong to DCX^+^ cells, as shown at a higher magnification in the upper right panel (b1). SGZ: subgranular zone; GCL: granule cell layer; BrdU, 5′-Bromo-2′-deoxyuridine; DCX, doublecortin. Scale bar: 50 µm (a-d); 10 µm (b1). e: quantification of DCX immunofluorescence in the SGZ of DG in different experimental conditions: control slices (n = 8) or in OGD slices in the absence (n = 8) or in the presence of BBG (10 µM, n = 4) or MRS2179 (10 µM, n = 6). Analyses were performed 24 hours from the end of OGD and at the corresponding times in control conditions. Each column represents the area of DCX immunofluorescence calculated using ImageJ on confocal acquisitions (number of pixels in thresholded images). Bars represent the mean±SEM. In parentheses is reported the number of slices investigated. **P<0.01 *vs* control and MRS2179 groups, One-way ANOVA followed by Newman–Keuls post hoc test.

In a parallel series of experiments, we investigated whether 9-min OGD modified cell proliferation and maturation in the SGZ, by performing immunohistochemical analysis for BrdU and DCX, respectively. The number of BrdU-immunoreactive (BrdU^+^) cells detected in the whole SGZ of DG of control slices was comparable at all times examined (Fig. 5a-c). The number of BrdU^+^ cells was not modified 3 hours after OGD, either in the absence or in the presence of BBG or MRS2179 (Fig. 5a). Conversely, 6 hours after the end of OGD, the number of BrdU^+^ cells was significantly decreased in comparison to controls (from 29.1±2.5 in controls, n = 8 to 17.4±2.0 after OGD, n = 10, *P*<0.01 Fig. 5b). This effect was completely antagonized by BBG, but not by MRS2179 (Fig. 6b). Indeed, the number of BrdU^+^ cells in the presence of MRS2179 (21.6±0.8, n = 6) was not statistically different from that found in OGD untreated slices (17.4±2.0, n = 10) but was significantly lower than that found in control slices (from 29.1±2.5 in controls, n = 8, to 21.6±0.8, n = 6 in MRS2179, *P*<0.05, Fig. 6b).

The number of BrdU^+^ cells in the SGZ returned to control levels at 24 hours from OGD, (29.5±3.3 in controls, n = 10; 28.9±2.8 after OGD, n = 14, not significant, Fig. 5c). In the presence of BBG no changes in the number of BrdU^+^ cells were detected at any time investigated (Fig. 5). Conversely, 24 hours from the end of OGD BrdU^+^ cells were significantly lower in the MRS2179-treated slices (14.3±0.8, n = 6, *P*<0.05, Fig. 5c).

In a parallel series of experiments we also evaluated the effects of BBG and MRS2179 on the number of BrdU^+^ cells in the SGZ under basal conditions. Each compound was applied for 24 min, corresponding to the time of drug application in OGD experiments. The number of BrdU^+^ cells was calculated after 6 or 24 hours from the end of drug application (S1 Fig.). No differences in the number of BrdU^+^ cells in control or in P2-antagonist treated slices were found. In particular, after 6 hours the number of BrdU^+^ cells was: 30.6±2.7, n = 9, in control; 30.3±1.9, n = 4 in the presence of 10 µM BBG; 29.0±1.0, n = 4 in the presence of 10 µM MRS2179 (S1a Fig.). Twenty-four hours after treatment the number of BrdU^+^ cells was: 30.5±2.9, n = 12, in control; 31.5±2.3, n = 4 in the presence of 10 µM BBG; 34.7±3.4, n = 4 in the presence of 10 µM MRS2179 (S1b Fig.).

The microtubule-associated protein DCX is a marker for cytoskeleton transiently expressed during adult neurogenesis. DCX-immunoreactive neuroblasts (DCX^+^) were clearly observed in the SGZ of control slices at 24 hours after slicing (Fig. 6a). Twenty-four hours after OGD the area of DCX immunofluorescence increased in comparison to controls (Fig. 6b,e). This phenomenon was still evident when OGD was applied in combination with BBG (Fig. 6c,e), but not in combination with MRS2179 (Fig. 6d,e). Quantitative analysis (Fig. 6e) confirmed that OGD significantly increased DCX protein immunofluorescence and this effect was blocked in the presence of MRS2179.

Higher magnification in Fig. 6b1 shows that DCX^+^ cells lay in the proximity of BrdU^+^ cells, indicating that BrdU^+^ nuclei belong to DCX^+^ cells. Similar observations were detectable in all the other experimental conditions (Fig. 6a-d) and are in agreement with our previous results [2].

## Discussion

In the present study we report that the selective P2X7R antagonist BBG and the selective P2Y1R antagonist MRS2179, applied before, during and after a severe OGD period, prevented the appearance of AD, an unequivocal sign of neuronal injury during ischemia, and allowed a significant recovery of fEPSP amplitude. These data support the notion that P2X7R and P2Y1R have a critical role in the progression of cell damage during ischemia in the DG. Moreover, our data demonstrate that severe OGD affects the number and maturation of proliferating cells in the SGZ and indicate that P2X7R and P2Y1R play a different role in these phenomena.

Both BBG and MRS2179 protect DG from the OGD-induced irreversible loss of synaptic transmission and in accordance protect from AD appearance. Therefore, both P2X7R and P2Y1R (activated during OGD by endogenously released ATP) exert a deleterious role on DG neurotransmission by participating in AD appearance.

The protective effect of BBG in blocking AD appearance and in preventing the irreversible loss of neurotransmission induced by severe OGD in DG is in agreement with our previous results obtained in the CA1 area [28], [46]. Several mechanisms might account for a deleterious effect of P2X7R in ischemia. A reasonable explanation is the inhibition of excess glutamate release as has been repeatedly shown in normoxic [53], [61] and ischemic hippocampal slices [62]. Transient stimulation of P2X7R with ATP induces Ca^2+^ influx into the cell [63], [64]. It has been also demonstrated that a sustained stimulation of P2X7Rs leads to conductance of moieties of up to 900 Da [65], generating continuous ATP and glutamate outflow, mainly described in astrocytes [66]. Thus, a prolonged activation of P2X7R, such as during a severe ischemic insult, causes an excess of intracellular Ca^2+^.

Also the protective effect of MRS2179 in blocking AD appearance and in preventing the irreversible loss of neurotransmission induced by severe OGD in DG is in agreement with our previous results obtained in the CA1 [28], [46]. It may be largely attributed to reduced intracellular Ca^2+^ levels and reduced glutamate outflow occurring after OGD. It has been demonstrated that the selective stimulation of P2Y1Rs on astrocytes *in vitro* induces glutamate release [67]–[69]. Moreover, harmful effects of P2Y1R during OGD may be attributable to a decrease of a well established adenosinergic A_1_ protective effect (for a review see in: [70]). It has been demonstrated that adenosine A_1_ receptor colocalizes with P2Y1R at glutamatergic synapses and surrounding astrocytes at the membrane level in rat hippocampus [71]. In this brain area P2Y1R stimulation impairs the potency of A_1_ receptor, whereas the stimulation of A_1_ receptors increased the functional responsiveness of P2Y1R [71].

Thus, prevention of synaptic failure and of AD development by P2Y1R and P2X7R antagonists after OGD in the DG may be attributed to direct or indirect (reducing glutamatergic activity) inhibition of intracellular Ca^2+^ loading in neurons and glial cells.

We also evaluated the effects of the P2 antagonists, applied alone or in combination, on AD development during 30 min OGD. Results indicate the each compound delays AD appearance, but we did not observe any additive effects when the antagonists were applied in combination. The lack of an addictive effect between BBG and MRS2179 in ameliorating OGD-induced AD development indicates that the two drugs work through a common pathway activated by P2X7R and P2Y1R modulation. Intracellular Ca^2+^ concentration might likely be a candidate since both P2Y1 and P2X7 receptors are known as efficient regulators of intracellular calcium concentrations [64], [72].

Observation that P2X7R antagonism does not modify synaptic transmission in the DG before OGD is in agreement with our previous results obtained in the CA1 region [46] indicating that P2X7R are not involved in low frequency-induced synaptic transmission in the DG under normoxia. In contrast to results observed in CA1 region, where MRS2179 inhibits synaptic transmission under normoxic conditions [28], [46], present results demonstrate that the block of P2Y1R does not affect DG neurotransmission. These data reveal that, in the DG, an endogenous activation of P2Y1R is not involved in low frequency-induced synaptic transmission under normoxia.

In agreement with our previous results [2], we report here the presence of BrdU- and DCX-labelled cells located in the SGZ, at the border between the hilus and the granule cell layer. Staining for DCX is proximal to BrdU^+^ nuclei, indicating that neuron committed cells are proliferating. Nine-min OGD rapidly induces a block of synaptic transmission that persists up to 24 hours ([2] and present work) and elicits a decrease in the number of BrdU^+^ cells in the SGZ 6 hours thereafter. This effect is likely due to acute excitotoxic damage induced by OGD. This is supported by observation that activation of NMDA receptors in the DG of adult rat rapidly decreases the number of proliferating cells [73]. The number of proliferating cells in the SGZ is restored 24 hours after the end of OGD, when a comparable number of BrdU^+^ cells between control and OGD slices has been found. Since BrdU^+^ was administered i.p. prior to isolation of hippocampal slices, the restoration of the number of proliferating cells can be explained by the increased proliferation of residual BrdU^+^ cells, with new daughter cells now exhibiting BrdU labeling.

In the presence of BBG a severe OGD never induces a reduction in BrdU^+^ cells at any time examined. Conversely, MRS2179 did not antagonize the reduction in the number of proliferating cells induced by OGD 6 hours after OGD and does not allow the restoration of the original pool of proliferating cells 24 hours after OGD. It is known that P2Y1R stimulation promotes cell proliferation in the SVZ niche in the mouse brain [74]. Moreover, a reduction of rapidly dividing BrdU^+^ cells has been observed in P2Y1 knock-out mice [74]. Finally, P2Y1Rs were shown to be involved also in the proliferation of adult SVZ-derived neurospheres [72]. On these bases, it is envisaged that the antagonism of P2Y1Rs, even if protective versus an acute ischemic damage on mature neurons, interferes with the trophic effect exerted by these receptors on neuroblasts at later times from OGD.

In the presence of MRS2179 or BBG under normoxic conditions, no modifications of the number of BrdU^+^ cells were found up to 24 hours after treatment. The reduction of BrdU^+^ cells observed in the presence of MRS2179 only after OGD might be due to strong stimulation of P2Y1 receptors during the ischemic insult. We hypothesize that under normoxic conditions in our model there is not sufficient P2Y1R stimulation (or P2Y1R density) able to activate trophic effects on proliferating cells.

In agreement with our previous results [2], 24 hours after 9-min OGD the DCX immunofluorescence is increased. DCX is a protein necessary for neuronal migration and its expression is required for the development of dendritic arborization, as demonstrated in primary hippocampal neurons [75]. Reduction of the expression levels of DCX in neuroblasts alters their migration [75]. In our experiments the increase of DCX after OGD may be considered an input for cell migration and maturation toward a neuronal phenotype. This effect was always evident when OGD was applied in combination with BBG but never in the presence of MRS2179. Since proliferation and differentiation of neural stem/progenitor cells is considered an important mechanism for neuronal restoration [1], [17], these results indicate that P2Y1R negatively influences neuronal maturation at later phases of OGD in the SGZ of DG.

In conclusion, the present data show, that by activation of P2X7R and P2Y1R, ATP (released in large amount during OGD) contributes to early ischemic brain damage by reducing synaptic activity in the perforant pathway, which is important for maintenance of DG cell proliferation [1], [73]. However, at later stages after OGD, P2Y1R might play an additional and opposite role in promoting cell proliferation and maturation in the DG.

## Supporting Information

S1 Fig **Quantification of BrdU^+^ cells in the SGZ of the DG at 6 and 24 hours after the end of BBG or MRS2179 treatment under basal, normoxic, conditions.** Each column shows the total number of BrdU^+^ cells in the SGZ after 6 hours (a) or 24 hours (b) after the end of drug application. Bars represent the mean±SEM. In parentheses is the number of slices investigated.
(TIF)
