# Supplementary figures and images for: The Selective Antagonism of P2X7 and P2Y1 Receptors Prevents Synaptic Failure and Affects Cell Proliferation Induced by Oxygen and Glucose Deprivation in Rat Dentate Gyrus
Source: PLoS One. 2014 Dec 19;9(12):e115273. doi: 10.1371/journal.pone.0115273 (PMC4272279; doi:10.1371/journal.pone.0115273)

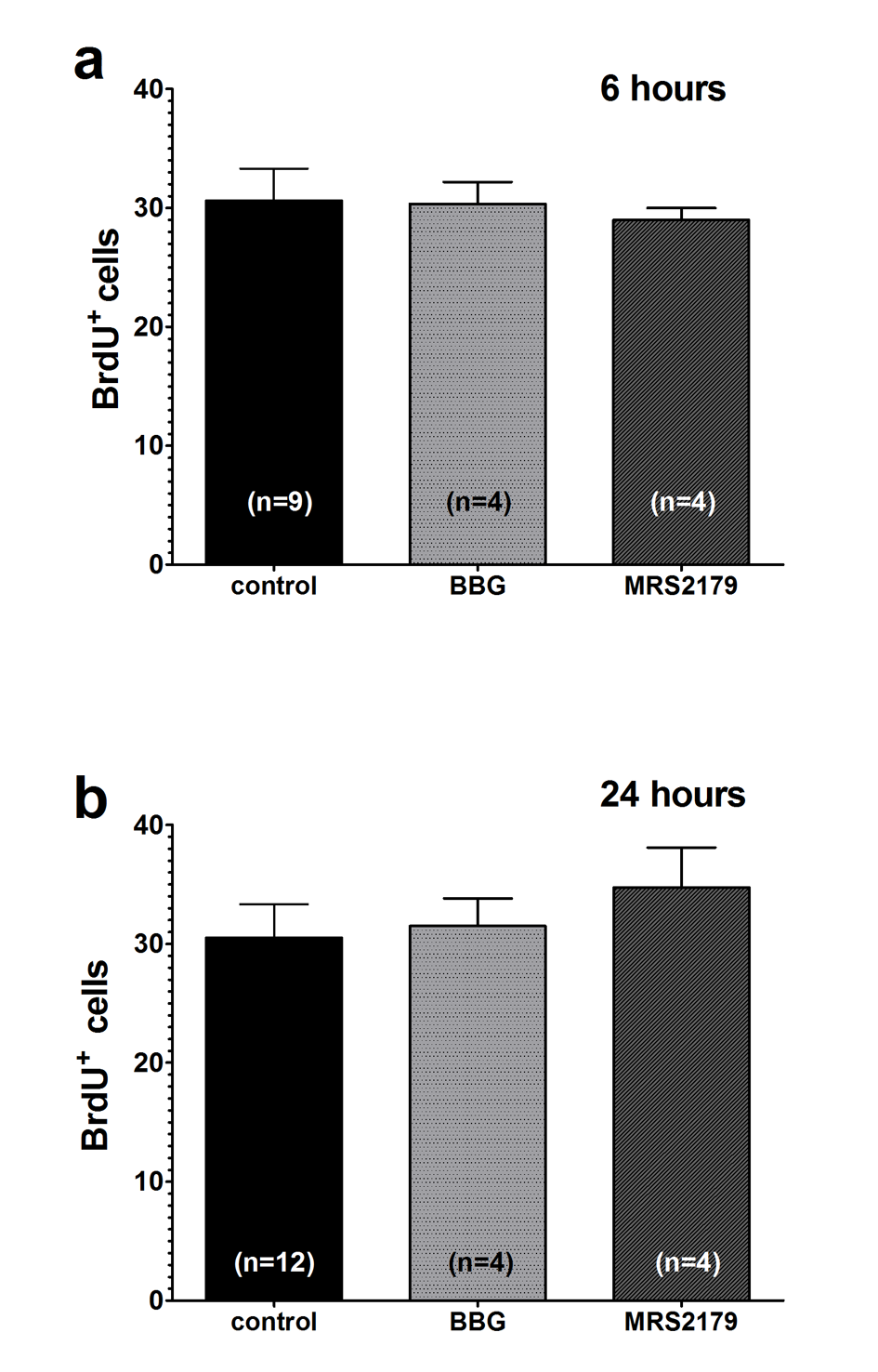

Supplement: S1 Fig — Quantification of BrdU+ cells in the SGZ of the DG at 6 and 24 hours after the end of BBG or MRS2179 treatment under basal, normoxic, conditions. Each column shows the total number of BrdU+ cells in the SGZ after 6 hours (a) or 24 hours (b) after the end of drug application. Bars represent the mean±SEM. In parentheses is the number of slices investigated. (TIF) [file pone.0115273.s001.tif]
